# Successful Reduction of Postoperative Chest Tube Duration and Length of Stay After Congenital Heart Surgery: A Multicenter Collaborative Improvement Project

**DOI:** 10.1161/JAHA.121.020730

**Published:** 2021-10-29

**Authors:** Katherine E. Bates, Chloe Connelly, Lara Khadr, Margaret Graupe, Anthony M. Hlavacek, Evonne Morell, Sara K. Pasquali, Jennifer L. Russell, Susan K. Schachtner, Courtney Strohacker, Ronn E. Tanel, Adam L. Ware, Sharyl Wooton, Nicolas L. Madsen, Alaina K. Kipps

**Affiliations:** ^1^ Congenital Heart Center University of Michigan C.S. Mott Children's Hospital Ann Arbor MI; ^2^ Department of Pediatrics University of Michigan Medical School Ann Arbor MI; ^3^ The Heart Institute Cincinnati Children’s Hospital Medical Center Cincinnati OH; ^4^ Department of Pediatrics University of Cincinnati School of Medicine Cincinnati OH; ^5^ Department of Pediatrics Children’s Heart Center Medical University of South Carolina Children’s Health Charleston SC; ^6^ Department of Pediatrics Heart Institute University of Pittsburgh Medical Center Children's Hospital of Pittsburgh Pittsburgh PA; ^7^ Department of Pediatrics Labatt Family Heart Centre The Hospital for Sick Children Toronto Ontario Canada; ^8^ Cardiac Center The Children’s Hospital of Philadelphia Philadelphia PA; ^9^ Department of Pediatrics Perelman School of Medicine University of Pennsylvania Philadelphia PA; ^10^ Pediatric Heart Center UCSF Benioff Children’s Hospital San Francisco CA; ^11^ Department of Pediatrics UCSF School of Medicine San Francisco CA; ^12^ Department of Pediatrics The Heart Center Primary Children’s Hospital Salt Lake City UT; ^13^ Anderson Center Cincinnati Children’s Hospital Medical Center Cincinnati OH; ^14^ Department of Pediatrics Betty Irene Moore Children's Heart Center Lucile Packard Children’s Hospital at Stanford Stanford School of Medicine Palo Alto CA

**Keywords:** cardiac surgical procedures, chest tubes, congenital, heart defects, length of stay, postoperative period, Quality and Outcomes

## Abstract

**Background:**

Congenital heart disease practices and outcomes vary significantly across centers, including postoperative chest tube (CT) management, which may impact postoperative length of stay (LOS). We used collaborative learning methods to determine whether centers could adapt and safely implement best practices for CT management, resulting in reduced postoperative CT duration and LOS.

**Methods and Results:**

Nine pediatric heart centers partnered together through 2 learning networks. Patients undergoing 1 of 9 benchmark congenital heart operations were included. Baseline data were collected from June 2017 to June 2018, and intervention‐phase data were collected from July 2018 to December 2019. Collaborative learning methods included review of best practices from a model center, regular data feedback, and quality improvement coaching. Center teams adapted CT removal practices (eg, timing, volume criteria) from the model center to their local resources, practices, and setting. Postoperative CT duration in hours and LOS in days were analyzed using statistical process control methodology. Overall, 2309 patients were included. Patient characteristics did not differ between the study and intervention phases. Statistical process control analysis showed an aggregate 15.6% decrease in geometric mean CT duration (72.6 hours at baseline to 61.3 hours during intervention) and a 9.8% reduction in geometric mean LOS (9.2 days at baseline to 8.3 days during intervention). Adverse events did not increase when comparing the baseline and intervention phases: CT replacement (1.8% versus 2.0%, *P*=0.56) and readmission for pleural effusion (0.4% versus 0.5%, *P*=0.29).

**Conclusions:**

We successfully lowered postoperative CT duration and observed an associated reduction in LOS across 9 centers using collaborative learning methodology.

**Nonstandard Abbreviations and Acronyms**
CT: chest tube
PAC^3^
: Pediatric Acute Care Cardiology Collaborative

**Clinical Perspective**

**What Is New?**

- Nine pediatric heart centers used collaborative learning methodology, including review of best practices from a model center, regular data feedback, and quality improvement coaching, to reduce variation in management of postoperative chest tubes following congenital heart disease surgery.
- As a group, these centers achieved statistically significant decreases in the duration of postoperative chest tubes and length of stay without any increases in adverse events, including need for replacement of chest tubes or hospital readmission because of pleural effusion.

**What Are the Clinical Implications?**

- Previously identified variation in chest tube duration was attributable primarily to variation in local chest tube management practices themselves rather than differences in patient populations or other local perioperative practices. Collaborative learning is an effective methodology to reduce variation across centers and ultimately improve patient outcomes.

Congenital heart disease (CHD) is the most common birth defect, representing the highest percentage of birth defect–associated hospital admissions and disproportionately high resource use across US children’s hospitals.^1^ Practices and outcomes in this patient population remain highly variable. One area of practice variability is the management of postoperative chest tubes (CTs), which are placed nearly uniformly following CHD surgery, the mainstay of treatment for many forms of CHD. The presence of a CT has many downstream consequences, including its impact on ambulation, analgesia, risk of infection, oral intake, and ultimately postoperative hospital length of stay (LOS). In the CHD population, LOS is a particularly important outcome given its known association with impaired neurodevelopment,^2^ resource use, and cost.

Our previous work demonstrated variation in CT management processes and outcomes across 9 congenital heart centers participating in the Pediatric Acute Care Cardiology Collaborative (PAC^3^) and the Pediatric Cardiac Critical Care Consortium (PC^4^).^3^ We identified 1 model center that had a shorter postoperative CT duration and shorter LOS for most procedures without evidence of higher rates of adverse events. At the outset of this study, it was unknown whether this center‐specific approach could be spread to other centers to decrease variation in CT management and thereby reduce postoperative CT duration and LOS.

Using collaborative learning methodology,^4^, ^5^, ^6^ we conducted a multicenter quality improvement project to reduce postoperative CT duration based on dissemination and implementation of the model center’s practices. The objective was to reduce the overall postoperative CT duration by 20%. We also studied the impact on postoperative LOS and adverse events (CT reinsertion and hospital readmission for pleural effusion).

## Methods

The data that support the findings of this study are available from the corresponding author upon reasonable request.

### Data Sources

Data from 2 learning networks (PAC^3^ and PC^4^) were used for this study. PAC^3^ focuses on quality improvement and research related to the acute care cardiology unit, defined as a hospital unit focused on caring for children with heart disease who do not require intensive care.^7^ PC^4^ aims to improve the quality of care and associated outcomes for patients in the pediatric cardiac intensive care unit. The PAC^3^ and PC^4^ registries share common terminology and definitions with applicable data points within the Society of Thoracic Surgeons Congenital Heart Surgery Database.^8^

Data linkage between PAC^3^ and PC^4^ was facilitated by Cardiac Networks United,^9^ an organization that supports integration of pediatric cardiovascular data and collaboration across networks to facilitate research and improvement. Data were linked using a common patient identifier and confirmed with operation type and date of procedure. PC^4^ data included baseline demographic information, diagnosis and operation type, perioperative information, and postoperative LOS. Postoperative LOS was defined as the time from date of surgery to date of discharge from the study center. From PAC^3^, we recorded variables specific to CT management, including CT duration, need for CT replacement during index hospitalization, and readmission because of pleural effusion within 7 days of discharge.

This study was approved by the Cincinnati Children’s Hospital Medical Center Institutional Review Board. A waiver of consent was granted.

### Study Population

All centers participating in both PAC^3^ and PC^4^ were invited to join the study. All index operations for patients who underwent 1 of the 10 benchmark operations defined by the Society of Thoracic Surgery^10^, ^11^ between June 2017 to June 2018 (baseline phase) and July 2018 to December 2019 (intervention phase) were eligible for inclusion. The benchmark operations are listed in Table 1 and span the spectrum of CHD complexity. We excluded patients who underwent the Fontan operation because none of the participating centers planned to apply the intervention to this unique patient population, because of significant differences in postoperative physiology and anticipated prolonged CT duration compared with the other benchmark operations.^3^ Therefore, patients undergoing 1 of the 9 remaining benchmark operations were analyzed. We also excluded patients requiring intervention for hemothorax and chylothorax, or who died before hospital discharge, because their management or outcomes would differ because of those conditions. Rates of hemothorax, chylothorax, and mortality were compared before applying these exclusion criteria to verify that important differences did not exist between the baseline and intervention phases. (Because of changes in the PC^4^ registry, the hemothorax variable was compared only for admissions before February 2019.). Patients with a second cardiothoracic operation during the same hospital admission or those who were transferred off the cardiology service for noncardiac management before discharge were excluded from the LOS outcome.

**Table 1:** Patient Characteristics at the Time of Index Operation

	Baseline, n=997	Intervention, n=1315	*P* value
Gestational age in weeks	38 [37, 39]*	38 [37, 39]^†^	0.78
Age, d, at surgery	117 [14, 182]	121 [16, 181]^‡^	0.91
Weight, kg, at surgery	5.1 [3.6, 6.7]	5.2 [3.7, 6.6]^‡^	0.57
No. of prior cardiothoracic surgical operations	0 [0, 0]	0 [0, 0]	0.21
Extracardiac anomaly	139 (13.9%)	205 (15.6%)^‡^	0.26
Genetic anomaly	163 (16.4%)	221 (16.8%)^‡^	0.75
Presence of any syndromes or syndromic abnormalities	217 (21.8%)	314 (23.9%)^‡^	0.22
Diagnosis of bronchopulmonary dysplasia	11 (1.1%)	9 (0.7%)^‡^	0.28
Preoperative morbidities			
Chest compressions with medications <48 h before surgery	9 (0.9%)	6 (0.5%)^‡^	0.19
Mechanical circulatory support	8 (0.8%)	5 (0.4%)^‡^	0.18
Shock at the time of surgery	12 (1.2%)	9 (0.7%)^‡^	0.19
Sepsis	5 (0.5%)	10 (0.8%)^‡^	0.44
Renal failure	11 (1.1%)	15 (1.1%)^‡^	0.93
Mechanical ventilation	175 (17.6%)	193 (14.7%)^‡^	0.06
Respiratory syncytial virus infection	2 (0.2%)	2 (0.2%)^‡^	1.00
Benchmark operation			
Ventricular septal defect repair	211 (21.2%)	276 (21.0%)	0.92
Off‐bypass coarctation repair	115 (11.5%)	158 (12.0%)	0.72
Tetralogy of Fallot repair	174 (17.5%)	241 (18.3%)	0.59
Bidirectional Glenn/HemiFontan	163 (16.4%)	231 (17.6%)	0.44
Arterial switch operation	79 (7.9%)	98 (7.5%)	0.67
Complete atrioventricular canal repair	106 (10.6%)	126 (9.6%)	0.41
Arterial switch operation and ventricular septal defect repair	30 (3.0%)	35 (2.7%)	0.62
Truncus arteriosus repair	20 (2.0%)	29 (2.2%)	0.74
Norwood operation	99 (9.9%)	121 (9.2%)	0.55
Bypass duration			
Cardiopulmonary bypass duration, min	101 [74, 143]^§^	102.5 [76, 142]^‖^	0.55
Adverse events			
Chest tube replacement	18 (1.8%)	26 (2.0%)	0.57
Readmission for pleural effusion	4 (0.4%)	6 (0.5%)	0.29

All values are expressed as median [Q1, Q3] or n (%).

* n=908 (gestational age is recorded only if patient is <365 days old on surgical date).

^†^ n=1213.

^‡^ n=1312.

^§^ n=868 (patients without any cardiopulmonary bypass not included).

^‖^ n=1134 (patients without any cardiopulmonary bypass not included).

### Intervention

Collaborative learning methods^4^, ^5^, ^6^ included transparent review of the model center’s best practices. Most prominently, although other participating centers typically removed CTs only when the volume of drainage dropped below a specified level (adjusted for patient weight) (Table 2), the model center’s practice was to remove CTs on the first postoperative day unless specific exclusion criteria were met (eg, concern for bleeding or thoracic duct injury).^3^ Additional collaborative learning methods included regular data feedback on practices and outcomes using a shared data‐reporting platform, monthly webinars to share intervention ideas and results, and quality improvement coaching. Each center was asked to adapt CT removal practices (eg, timing, volume criteria) from the model center to their own local resources, practices, and setting to reduce CT duration. Each center’s individual approach was recorded by the study team. Centers were invited to participate in the intervention phase at a PAC^3^ meeting held in May 2018, with the resulting intervention phase beginning in July 2018.

**Table 2:** Approach to Chest Tube Management and Implementation Strategies Across Centers

Domain	Chest tube removal strategies	Model	Site A	Site B	Site C	Site D	Site E	Site F	Site G	Site H
Timing of implementation	Changes made by October 2018									
	Changes made after October 2018									
	No changes made									
Strategy before implementation	<3 mL/kg per 24 h				1V and 2V	1V	1V and 2V			1V and 2V
	3–6 mL/kg per 24 h		1V and 2V	1V and 2V		2V		1V and 2V	1V and 2V	
	Aim for POD 1	1V and 2V								
Strategy for biventricular procedures after implementation	Goal to remove tubes on POD 1									
	Goal to remove tubes on POD 2									
	Volume criteria: remove when <10 mL/kg per 24 h (or as noted)			<10 mL/kg per 12 h or <200 mL/12 h if >40 kg	If not removed POD 2 → volume criteria	If not removed POD 1 → volume criteria	<8 mL/kg per 24 h or <250 mL/ 24 h if >30 kg		If not removed POD 1 → volume criteria	
	Age limit for inclusion			Neonates only if chest closed, <6 mL/kg per 24 h		Neonates only if chest closed and right atrial line out	>4 mo old			
	Strategy implemented for all biventricular surgeries (in addition to STS benchmark procedures)						All patients without prior sternotomy	All except for TOF		
Strategy for univentricular procedures after implementation	Goal to remove tubes on POD 1	S1P and SCPC	SCPC					SCPC		
	Goal to remove tubes on POD 2				S1P and SCPC					
	Volume criteria: remove when <10 mL/kg per 24 h (or as noted)			SCPC (S1P: <6 mL/kg per 24 h)		S1P and SCPC: <6 mL/kg per 24 h			SCPC (S1P if chest closed: <6 mL/kg per 24 h)	
	No change made for 1V population									

Gray shading indicates timing of implementation and strategies chosen by each center.

1V indicates univentricular procedures; 2V, biventricular procedures; POD, postoperative day; S1P, stage 1 palliation with closed chest; SCPC, superior cavopulmonary connection; STS, Society Thoracic Surgeons; and TOF, tetralogy of Fallot.

### Outcomes

The primary outcome was CT duration in hours, calculated as the difference between the time of postoperative cardiac intensive care unit admission and the time when the final perioperative CT was removed. Perioperative CTs were defined as all CTs placed during surgery (intraoperative) or while the original CTs from surgery were still in place (postoperative). Secondary outcomes included total postoperative LOS measured in days, calculated as the difference between the time of postoperative cardiac intensive care unit admission and the day of hospital discharge. Recorded adverse events included the frequency of CT replacement after the final perioperative CT was removed, and hospital readmission within 7 days of discharge because of pleural effusion.

### Statistical Analysis

We used statistical process control methodology to examine CT duration and LOS over time. Statistical process control is commonly used to assess changes in health care settings because it accounts for random or common‐cause variation in outcomes and is more sensitive to small changes over time as compared with traditional statistical methods. Statistical process control uses a graphical display, allowing for annotation of the timing of interventions.^12^, ^13^ Both CT duration and postoperative LOS were plotted on Xbar and S charts after performing a log transformation on the data given the positive skew distribution of both outcomes.^13^, ^14^ This 2‐part control chart shows both monthly means and monthly standard deviation on the same *x* axis in the Xbar and S sections of the chart, respectively. The centerline or mean of both charts (given the log transformation, the centerline represents the geometric mean in this analysis) are recalculated when sustained special cause variation is detected in either chart. Given the timing of our network discussions outlined above, we set the baseline centerline and control limits using data through the end of the baseline phase (May 2018) and then looked for special cause variation signals relative to this baseline.^13^ We used standardly accepted rules for identifying special cause variation, including observing at least 8 consecutive points above or below the centerline, a probability‐based rule that roughly corresponds to a *P*<0.01.^14^ For ease of interpretation, the data, control limits, and centerline were transformed back into the original units for display, and only the Xbar chart is shown in Results (paired Xbar and S charts shown in Figures S1 and S2).^13^

Traditional statistical methods were used to compare patient demographics between the baseline and intervention phases. Categorical variables were summarized as frequency (percentage) and compared using χ^2^ or Fisher exact χ^2^ tests. Continuous variables were summarized as median (interquartile range) and compared using the Wilcoxon rank sum test.

As is conventional for studies of multicenter collaborative quality improvement projects,^15^, ^16^, ^17^ we analyzed the data for all centers, including the model site, as a group. We also analyzed the CT duration and postoperative hospital LOS in 2 subsets of patients in a post hoc analysis: (1) a group of 4 early‐adopter centers that reported successful early implementation of changes to their CT management practices and (2) the 2 control centers that submitted data but reported no changes to their CT management processes.

For all statistical tests, a *P* value of <0.05 was considered statistically significant. Analyses were performed using SAS 9.4 (SAS Institute, Cary, NC).

## Results

A total of 2309 patients were enrolled from 9 PAC^3^ and PC^4^ centers that agreed to participate. There were no significant differences in patient characteristics between the baseline and intervention phase cohorts (Table 1). There were no significant differences between the baseline and intervention phases in overall rates of chylothorax (6.0% versus 6.5%, *P*=0.59), hemothorax (0.3% versus 0.3%, *P*=1.0), or mortality (2.1% versus 2.2%, *P*=0.70).

Centers chose a variety of approaches to adapt the model center’s CT management strategy, with variation in the target postoperative day and specified CT output volumes required for removal, but all strategies focused on removing CTs earlier in the postoperative course. Table 2 summarizes each center’s approach to intervention, reflecting all changes that occurred before an in‐person collaborative learning meeting convened in May 2019 (month 11 of intervention phase).

### CT Duration

**Figure 1:**
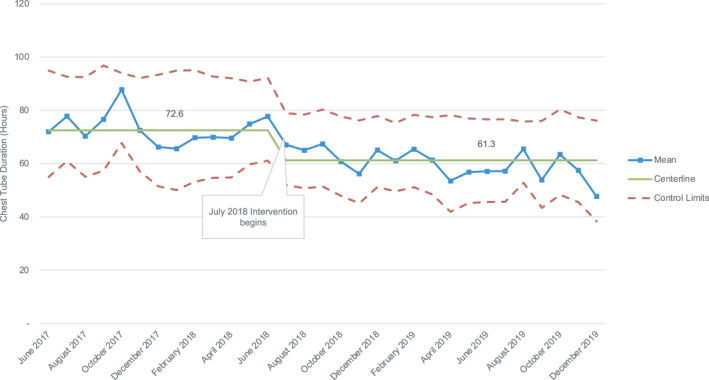
Xbar statistical process control chart for chest tube duration. There was a 15.6% decrease in the centerline from 72.6 to 61.3 hours.

Using statistical process control, special cause variation in the mean monthly CT duration was noted on the Xbar chart with >8 points below the centerline, coinciding with the start of the intervention phase in July 2018. When the centerline and limits were recalculated, there was a decrease in the centerline CT duration from 72.6 to 61.3 hours, representing a 15.6% reduction (Figure 1). There was no significant decrease in the control center subgroup.

### LOS and Adverse Events

**Figure 2:**
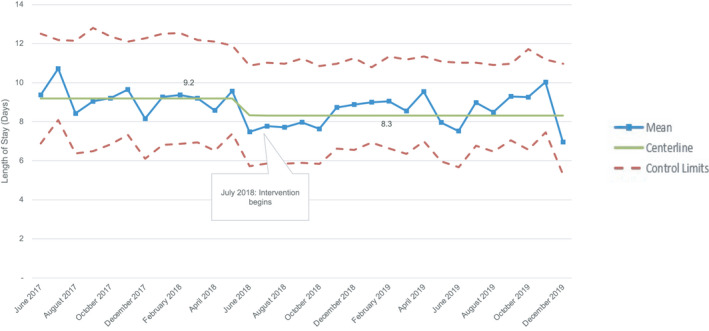
Xbar statistical process control chart for postoperative length of stay. There was a 9.8% decrease in the centerline from 9.2 to 8.3 days.

When examining LOS, there was special cause variation identified in the Xbar chart with >8 points below the centerline starting in June 2018. When the centerline and control limits were recalculated, there was a 9.8% decrease in the centerline LOS from 9.2 to 8.3 days (Figure 2). As seen with CT duration, there was no significant difference when the control center subgroup was analyzed separately. There was no difference between baseline and intervention phases in regard to frequency of CT replacement or hospital readmissions within 7 days because of pleural effusion (Table 1).

### Early‐Adopter Centers

The 4 early‐adopter centers enrolled a total of 917 patients. Early‐adopter centers varied in their approach to removing CTs earlier but were similar in timing of implementation. Each of the 4 early‐adopter sites reported that their first meetings with pediatric cardiothoracic surgeons occurred between June 2018 and August 2018 and that their first interventions began by October 2018. This early engagement with multiple stakeholders and commitment to removing CTs earlier differed significantly from the remainder of the participating centers (Table 2). Center leads for 3 of the 4 early‐adopter centers were also leaders of the overall PAC^3^ effort, which may have positively influenced the process at their centers.

**Figure 3:**
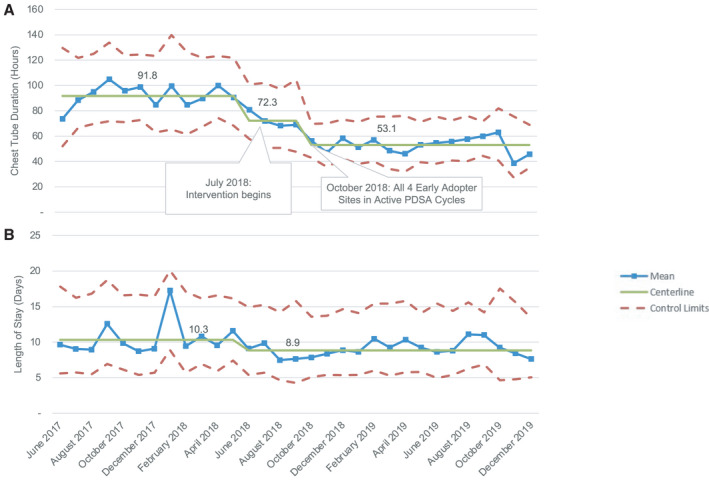
Xbar statistical process control charts for chest tube duration (**A**) and postoperative length of stay (**B**) in the 4 early‐adopter centers. **A**, There were 2 decreases in centerline from 91.8 to 72.3 hours and then to 53.1 hours for a total decrease of 42%. **B**, There was a 14.6% decrease in the centerline from 10.3 to 8.8 days. PDSA indicates, Plan‐Do‐Study‐Act.

Statistical process control analysis showed multiple special cause variations in the early‐adopter Xbar chart, starting with an initial decrease in the centerline from 91.8 to 72.3 hours (June 2018–September 2018) and then a further decrease after October 2018 to a centerline of 53.1 hours, for a total decrease of 42%. Early adopters also achieved substantial reductions in LOS with a 14.6% decrease in the centerline from 10.3 to 8.8 days (Figure 3). These decreases were the most significant seen among the 9 centers participating.

## Discussion

We successfully reduced postoperative CT duration and observed an associated decrease in LOS across 9 centers participating in a collaborative learning improvement project. Importantly, the CT duration reduction was achieved without increases in CT replacement or hospital readmission because of pleural effusion, indicating that there were no measurable adverse events from these practice changes. Additionally, we identified greater reductions in postoperative CT duration and LOS in 4 early‐adopter centers, suggesting that there may be additional opportunity for improvement and decreased variability across the other participating pediatric heart centers over time as implementation is fully realized.

The successful reduction of CT duration across participating centers through dissemination and modified implementation of model center practices suggests that previously noted variation in CT outcomes was attributable primarily to variation in local CT management practices themselves rather than differences in patient populations or other local perioperative practices. In a previous survey of PAC^3^ centers, we found that CT management practices were primarily based on local guidelines instituted without the benefit of data, often with internal variation dependent on the shifting of different clinicians and their application of different guidelines.^18^

One recently published pediatric single‐center study reported a successful reduction in CT duration through use of a standardized management algorithm,^19^ but to our knowledge, this is the first multicenter study reporting a reduction in CT duration and LOS. Interestingly, although the model center’s practices were shared transparently, none of the centers matched the model center’s practice exactly during the intervention phase. The overall effort was successful despite the significant variation in the centers’ approaches to removing CTs earlier, including types of operations included, goal postoperative day for removal, volume criteria for removal, and the timing of the intervention at their center. As a result, and as exemplified by the early‐adopter centers that may have achieved a more significant CT duration reduction simply by starting earlier, there remains opportunity to reduce variation in CT management to improve patient outcomes. In recognition of this potential for additional improvement, many of these participating centers, including the early‐adopter centers, have continued to modify their CT management practices to further reduce CT duration and LOS.

The observed reduction in postoperative LOS supports our hypothesis that CT duration is related to LOS. Importantly, only 1 participating early‐adopter center reported an additional effort to reduce LOS during the study period; however, interventions related to this effort began in November 2019, many months after we identified special cause reduction in LOS. Although decreased CT duration appears associated with lower LOS in our study, there may be other contributing factors, particularly for those patients with prolonged LOS.^19^, ^20^ We appreciate that the relationship between CT duration and LOS may vary depending on operation and patient age. For example, the LOS for a newborn following a Norwood operation is likely impacted more by the need to establish enteral feeding and preparation for interstage home monitoring than by CT duration, given the large gap between CT duration and LOS shown in our previous work.^3^ In contrast, the postoperative LOS for an older infant with established feeding undergoing a ventricular septal defect repair will be more directly impacted by CT duration. Of note, there were no differences between the baseline and intervention cohorts in benchmark surgery types or other operative risk factors such as weight, age, and bypass time (Table 1), which might otherwise explain differences in LOS.

Finally, the magnitude of the observed LOS reduction was surprising, particularly because most centers focused interventions on lower complexity operations with shorter LOS than the more complex CHD surgeries.^3^ This finding supports a recent study by Pasquali et al, which suggests that significant improvements in CHD care might be achieved by focusing on higher volume and lower complexity operations, in contrast to many previous quality improvement efforts that have focused on rarer and more complex conditions.^21^ Although not directly measured as part of this study, it is possible that earlier removal of CTs reduces the need for analgesia medications and promotes early ambulation, thereby reducing side effects from narcotics and bed rest, which might further reduce LOS.

Our findings support the use of collaborative learning methodology, particularly when a gold standard or model site with best practices can be identified as a guide for other centers. This approach is well described by the Institute for Healthcare Improvement’s Breakthrough Series model^22^; however, our approach differed because it was successful in the absence of an evidence‐based best practice given the lack of clinical trials on CT management or outcomes.^18^ Our approach also differed from the Pediatric Heart Network’s successful Collaborative Learning Study focused on early extubation in patients undergoing CHD surgery, because each center developed its own approach to remove CTs earlier, using the model site as inspiration rather than codesigning a protocol based exactly on the model site’s practice.^6^ This flexible approach to collaborative learning, using identification of variation and allowing for customization and experimentation at the individual center level, may have contributed to the successful implementation^23^ and could prove to be particularly useful in fields with relatively few evidence‐based practices, as is often the case in CHD care and other pediatric populations. Our approach aligns more closely with the Anderson Center Learning Network model, which has been successfully used in several pediatric fields.^16^ However, this flexible approach may also have limited the impact of this effort, because some centers’ approaches were significantly less aggressive than the model center. Other factors that may have contributed to the success of the CT intervention include its relative simplicity, trialability on a single patient or group of patients, and relative advantage over previous practice. Furthermore, the collaborative learning structure built active engagement among participants and included quality improvement coaching and tools to assist with the process of implementation at each center, which also likely contributed to success^23^ and may increase the sustainability of these interventions over the long term.^20^, ^21^

Future directions of this work include expanding the multicenter collaborative learning structure to an additional 9 PAC^3^ and PC^4^ sites that began collecting baseline data in May 2019. All the while, the original centers continue to participate in collaborative learning activities, further refining their CT management processes and serving as mentors for these centers that have more recently joined the effort. Furthermore, PAC^3^ plans to use similar structures for new collaborative learning initiatives based on early work with a smaller group of engaged centers followed by intentional spread to a larger group. Finally, we plan to assess sustainability of the CT interventions following completion of the active collaborative learning phase, using the PAC^3^ and PC^4^ registries as a mechanism for long‐term data collection.

### Limitations

Findings from this study may not be generalizable to other centers, particularly centers that elect to implement changes outside of a collaborative learning structure. These findings also may not be generalizable to other pediatric cardiothoracic operations, particularly for populations of patients with defining features such as Fontan circulation, the group intentionally excluded from this study. Given the multitude of factors that impact LOS, it is possible that the decrease in LOS that we observed was not caused by the decrease in CT duration. Finally, because we continue to support an active collaborative learning effort, it is not yet possible to assess the sustainability of the CT interventions after conclusion of the effort. However, we believe that the same factors that contributed to the success of the intervention and our plan to monitor CT duration as a part of the network registries will increase the likelihood of sustained improvement.^24^, ^25^

## Conclusions

We successfully lowered postoperative CT duration and observed an associated reduction in LOS across 9 centers participating in PAC^3^ and PC^4^ using collaborative learning methodology. Future directions include spreading this intervention to additional centers and evaluating the sustainability of these reductions across all participating sites.

## Sources of Funding

PC^4^ is supported by funding from the University of Michigan Congenital Heart Center, Champs for Mott, and the Michigan Institute for Clinical & Health Research (National Institutes of Health/National Center for Advancing Translational Sciences, UL1TR002240). PAC^3^ is supported by the Heart Institute at Cincinnati Children’s Hospital. Cardiac Networks United is supported by the Children’s Heart Foundation, in addition to receiving support from the University of Michigan Congenital Heart Center and the Heart Institute at Cincinnati Children’s Hospital.

## Disclosures

None.

## Supporting information

Figures S1–S2
