# Supplementary material for: Successful Reduction of Postoperative Chest Tube Duration and Length of Stay After Congenital Heart Surgery: A Multicenter Collaborative Improvement Project
Source: J Am Heart Assoc. 2021 Oct 29;10(21):e020730. doi: 10.1161/JAHA.121.020730 (PMC8751825; doi:10.1161/JAHA.121.020730)
Supplement: Supplementary file 1 — Figures S1–S2 [file JAH3-10-e020730-s001.pdf]

Supplemental Material

Figure S1. Xbar S statistical process control chart for chest tube duration.

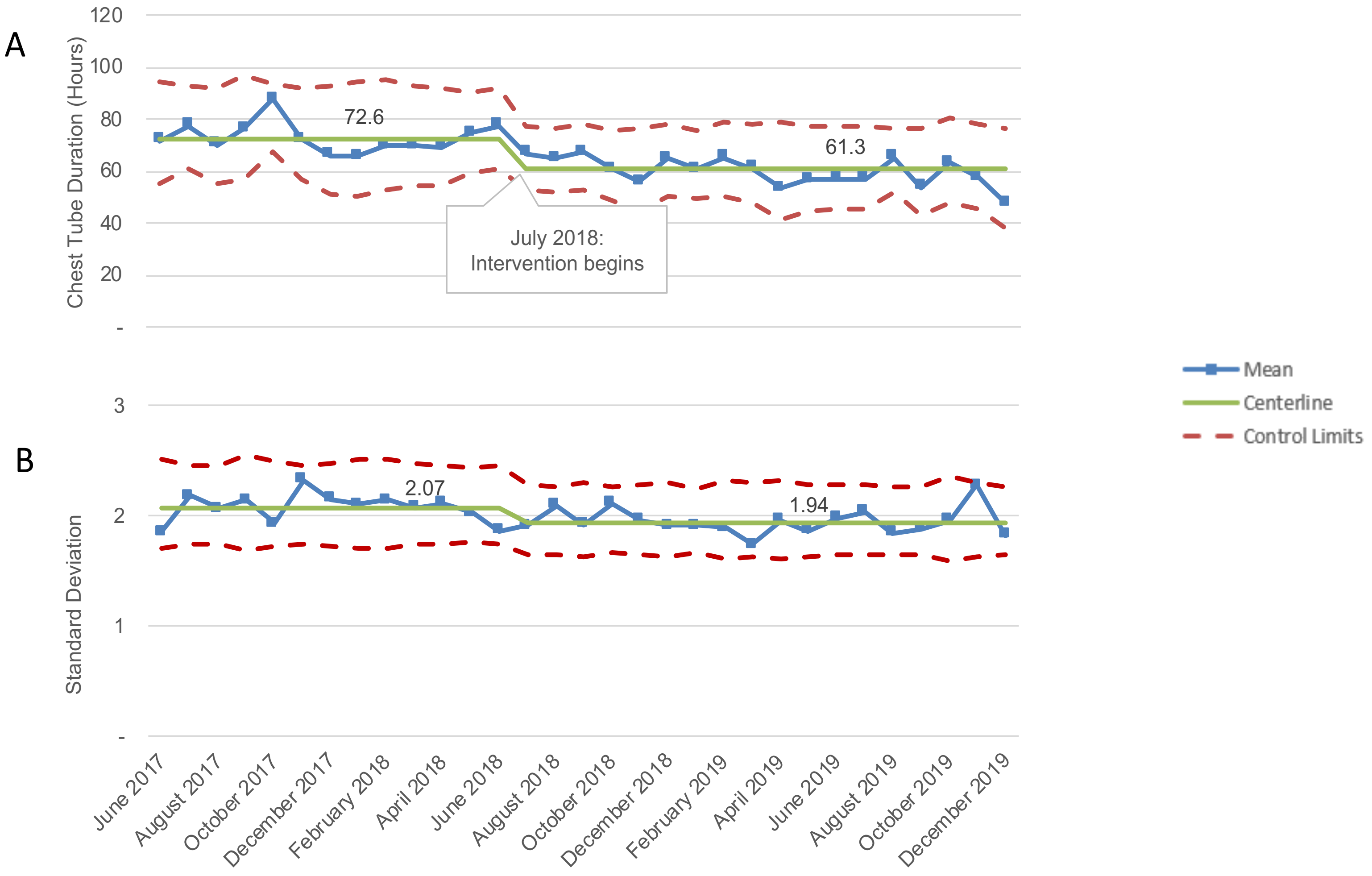

A, Xbar chart. There was a 15.6% decrease in the centerline from 72.6 hours to 61.3 hours. B, S chart. There was a 9.5% decrease in the centerline from 2.1 to 1.9.

Figure S2. Xbar S statistical process control chart for postoperative length of stay.

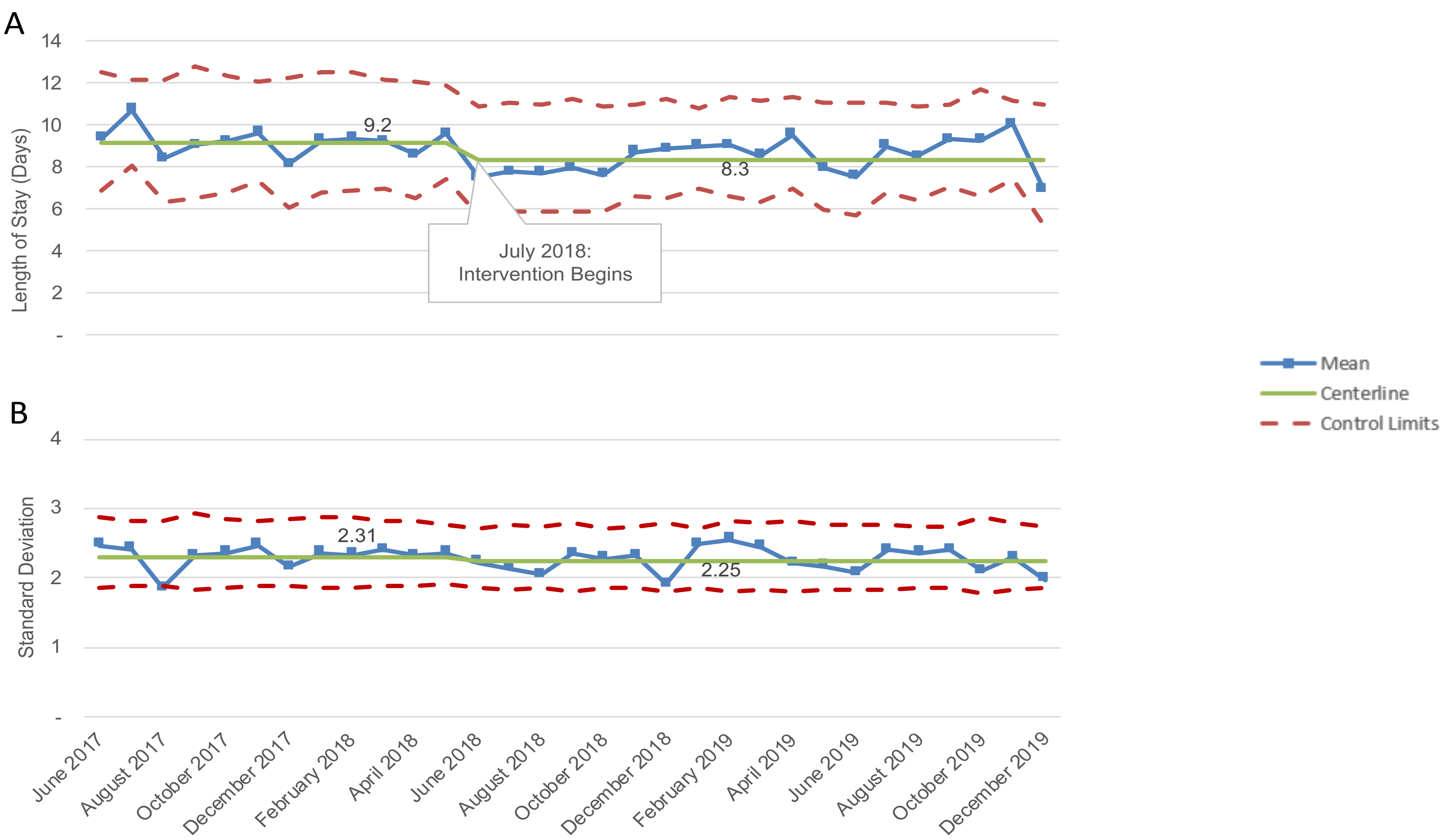

A, Xbar chart. There was a 9.8% decrease in the centerline from 9.2 days to 8.3 days. B, S chart. There was a 2.6% decrease in the centerline from 2.31 to 2.25.
